# Parasitic infections in relation to practices and knowledge in a rural village in Northern Thailand with emphasis on fish-borne trematode infection

**DOI:** 10.1017/S0950268818002996

**Published:** 2018-11-15

**Authors:** K. Chaisiri, C. Jollivet, P. Della Rossa, S. Sanguankiat, D. Wattanakulpanich, C. Lajaunie, A. Binot, M. Tanita, S. Rattanapikul, D. Sutdan, S. Morand, A. Ribas

**Affiliations:** 1 Faculty of Tropical Medicine, Department of Helminthology, Mahidol University, Bangkok, Thailand; 2 CIRAD ASTRE, Kasetsart University, Bangkok, Thailand; 3 CIRAD, UPR HortSys, F-97285 Le Lamentin, Martinique, France; 4 INSERM, Ceric-DICE CNRS, Aix-Marseille University, Marseille, France; 5 ASTRE, INRA, CIRAD, Univ Montpellier, Montpellier, France; 6 Saen Thong Health Promoting Hospital, Tha Wang Pha, Nan, Thailand; 7 Tha Wang Pha Hospital, Tha Wang Pha, Nan, Thailand; 8 CNRS ISEM – CIRAD ASTRE, Faculty of Veterinary Technology, Kasetsart University, Bangkok, Thailand; 9 Section of Parasitology, Faculty of Pharmacy and Food Sciences, Department of Biology, Healthcare and the Environment, University of Barcelona, Barcelona, Spain

**Keywords:** Fish-borne trematode, health education, health risk factors, helminths, participatory mapping, protists, Thailand

## Abstract

The present study integrates several aspects of a parasitological survey in a rural community village combining community knowledge of parasites, their potential transmission routes and health risk factors. A rural community located in Northern Thailand was surveyed for intestinal parasites, and an overall prevalence of 45.2% for helminths and 4.8% for protozoan infections was identified. Socio-demographic characteristics, customs and perceptions were compiled using individual questionnaires and interviews for participants surveyed for parasitic screening. The results allowed us to determine the knowledge and perception of local people concerning helminthic infection and transmission. Despite the fact that the participants in this community were aware of parasitic transmission routes, their widespread custom of eating raw fish and meat render the reduction of helminthiasis difficult. A detailed study on the infection of fish-borne parasitic trematodes, the most prevalent helminth, allowed us to determine that the distance from a given household to the river is a determinant of infection intensity. Health education activities organised in the local community resulted in a change in perception of risks associated with parasite transmission.

## Introduction

Southeast Asia is the region with the highest prevalence of soil-transmitted helminthic infections reported over the last few decades [1]. *Opisthorchis viverrini*, a fish-borne trematode, is known to be one of the factors that may lead to bile duct cancer (cholangiocarcinoma), which is endemic to Southeast Asia [2], and other helminths such as *Taenia* spp. are also widespread among Southeast Asian populations [3]. Intestinal parasitic infections (helminths and protists) transmitted via the faecal–oral route are also of importance in this region due to the tropical environmental conditions that facilitate the maintenance of their life cycles. An overall decline in the prevalence of helminthiasis has been observed in Thailand over the last few decades, from 54.7% in 1980–1981 [4] to 18.1% in 2009 when the last national survey was conducted [5]. However, according to this most recent survey, pockets of high infection still remain, particularly of *O. viverrini*, hookworms and *Strongyloides stercoralis* in the north and northeast of Thailand [5]. Changes in the prevalence and incidence of pathogenic intestinal protists have not been estimated. However, the conditions and human behaviours that favour intestinal protists of medical importance are common to several helminth species; consequently, a decrease in the infection rates of many protists may be expected with a general approach.

The National Health Assembly (NHA) of Thailand, an institution representing the government sector, civil society and the academic sector, passed a resolution in 2014 for the elimination of liver fluke (opisthorchiasis) and bile duct cancer (cholangiocarcinoma) in humans. The resolution, acknowledging the importance of the incidence and mortality of bile duct cancer, insisted on the fact that ‘the action taken must be integrated and cover all risk factors concerned, behavioural, environmental and cultural’ [6].

In Thailand, the overall poverty rate has continuously declined over the past 40 years, as shown by the increase in the Human Development Index from 0.574 in 1990 to 0.74 in 2015 [7]. Poverty is more prevalent in rural than urban areas, despite Thailand's universal healthcare system now providing coverage to 98% of the population [8]. Notable parasitic incidences and prevalences are still recorded in parasitological surveys in rural communities [9]. The identification of parasitological risk factors is important to explain prevailing parasitic infections in rural communities, which is one of the objectives of the present study. Although numerous parasitological studies have been conducted in rural Thai communities, the identification of risk factors is mostly limited to studies of single parasites such as *Opisthorchis* [10] or hookworms [11], despite the fact that rural communities are affected by multiparasitism (helminths and protists). The lack of studies covering the complete range of parasitic infections and including some risk factor analysis has been noted in other studies concerning Southeast Asian countries, such as a report of *Clonorchis sinensis* studied in Vietnam [12].

A community-based study at the level of individual villager could potentially explore parasitic transmission and help to understand local perceptions of parasites, which may reveal some crucial factors influencing parasitic transmission. Thus, the aim of the present study was to acquire a better understanding of helminthic and protist infections in a rural community in Northern Thailand in relation to socio-demographics and the attitudes, customs, perceptions and knowledge of locals, as assessed by questionnaires, interviews and participatory methods.

## Methods

### Study area

Individual participants inhabit Huay Muang village, located in Tha Wang Pha District (Nan Province), Northern Thailand. The centre of the village is situated at latitude 19.14°N and longitude 100.72°E at an elevation of around 300 m and is bordered by the river Rim. It is an ideal location for study because it is near the border with Laos in an area particularly rife with parasitic infections [13]. The population of the village was 452 inhabitants, according to the Subdistrict Administration Office (SAO). This village has a predominantly agricultural way of life based on rice and maize cultivation, together with teak plantations. Agricultural plots are either on the edge of the river, permitting effective irrigation of the fields, and therefore flooding, or situated on slopes. The owners of fields along the river benefit from the high fertility of these plots. Demand for maize has driven the exploitation of slopes, potentially increasing soil erosion [14].

### Human stool collection and intestinal parasitic examination

In November 2012, a faecal sample (around 5 g) was collected from each participant in a plastic container which had been distributed to villagers with help from local health volunteers. The samples were brought to the field laboratory for further examination within the same day. The geographical coordinates of each household were recorded using a Garmin Montana 600 GPS device (Garmin International Inc., Olathe, KS, USA).

The faecal samples were first checked for parasitic infections using the direct smear technique to screen for helminths and protozoa. Lugol's iodine was added to simple smears as a stain to better visualise and identify protozoa. Intestinal protozoan identification was performed by consulting the taxonomic literature [15, 16]. Subsequently, a concentration method, a modified Katz thick smear technique [17], was followed to identify and quantify helminthic eggs or larvae present in faecal samples. The slides were coated with faecal samples (around 43 mg) sieved through a wire-net filter and then covered with a cellophane strip pre-soaked in Katz's solution (glycerine–malachite green solution). The prepared slides were left at room temperature for 30 min to allow the faecal matter to dry and clear, then the slides were examined and all helminthic eggs were counted under a light microscope by experienced parasitologists. Duplications of both the modified Katz thick smear and the simple smear were performed for each faecal sample. To assess the intensity of helminthic infection, the number of eggs per gram was calculated as described previously [17].

### Ethical considerations

The study procedures concerning human sample collection, laboratory investigation, interviews and questionnaires were reviewed and approved by the Ethical Committee of the Faculty of Tropical Medicine, Mahidol University (Bangkok, Thailand), Document No. 0517.1116/661. First, the investigation team conducted a meeting with local public health officers, the village chief and local health volunteers to inform people of the purpose and details of the research study. Then, public loudspeaker broadcasting was used to notify all households of the research goals in the local language. All participants who agreed to join the study were asked to read the participant information sheet explaining the objectives, procedures, possible risks and benefits of the research project. Consent/assent forms were signed by either participants themselves or the parents of child participants prior to faecal sample submission and acquisition of personal information through interviews and questionnaires. The age range of participants was from 10 to 102 years old. All participants infected with intestinal parasites, including helminths and protists, were informed individually of the diagnostic results and offered treatment by qualified clinicians from Tha Wang Pha District Hospital (Nan Province) and the Faculty of Tropical Medicine, Mahidol University. All infected participants were given deworming drugs: a single dose of praziquantel (40 mg/kg) was administered for fluke (fish-borne trematodes and *Paragonimus*) and tapeworm (*Taenia*) infection, while albendazole (400 mg tablet, single dose) was prescribed for nematode infection (*S. stercoralis* and hookworms).

### Interviews and questionnaires

The standardised questionnaires distributed to the inhabitants of Huay Muang village were carried out by the staff of the Faculty of Tropical Medicine, Mahidol University and validated by the Ethical Committee of Mahidol University, Document No. 0517.1116/661. Data were collected in Thai by the team at the Saen Thong primary care health unit (PCU), Tha Wang Pha District, Nan Province, and translated into English. These questionnaires provided individual data regarding participants’ age, gender, level of education, profession, eating habits and knowledge of different types of parasites (by asking about the names of helminth species and their routes of transmission). The questionnaires were completed by 198 of the 228 villagers who participated in parasitic screening. The acquired data were merged with personal and demographic information (e.g. sex, age, address, educational level and occupation) extracted from the PCU records. Houses were geo-localised, and the distance (in metres) from each house to the river Rim was determined using Google Earth [18].

### Participatory mapping

In December 2012, participatory mapping was used as a way to collect data in a collective manner [19]. During the mapping session, a collective interview was conducted involving 30 people who were among the 200 villagers who had participated in the parasitic screening. The group included both men and women, traditional healers, medical volunteers and village leader delegates. A satellite image of the village of Huay Muang and its surroundings was provided to the focus group during two participatory sessions and was used to draw a map locating flooding events and places perceived as high risk.

### Statistical methods

#### Multivariate analyses

First, we used principal component analyses to explore (1) individual parasitic infections (helminths and protozoa) and (2) the results of the individual questionnaires. This statistical exploration helped to quantify the homogeneity or the disparity in the infections and the responses to questionnaires by exploring the percentage of variance explained by the first two axes of each analysis, with a low percentage of variance suggesting low homogeneity among individual villagers (a higher number of axes would be necessary to capture the whole variance). We used the princomp function of the FactoMineR package, with the data centred and scaled. These analyses helped to compare the results of the present study with those of a previous study [20].

Second, canonical correspondence analysis was performed to test the statistical association among the individual data for the two sets of variables. Wilk's lambda (Wilk's L) was used to test how well each canonical dimension contributed to the statistical multivariate model. The scale of Wilk's L ranges from 0 (full discrimination) to 1 (no discrimination). These analyses were performed using MASS version 7.3–45 [21], FactorMineR and CCA version 1–2 embedded in R [22].

#### Univariate analyses

Logistic regressions have previously been used by different studies to identify risk factors for parasitic infection [23, 24]. A negative binomial model was used to overcome the overdispersion of the fish-borne trematode, *O. viverrini*-like egg count (while checking that the latter type had a better Akaike information criterion (AIC) than the Poisson model). The location of the river was introduced as distance in metres. Potential risk factors were selected at an *α* risk of 20% in univariate. A stepwise selection method based on minimisation of the AIC was then used to suppress variables that did not significantly explain the rate of infection.

We also verified the non-nullity of the coefficients using the Wald test (*P*-value <5%). The interactions were tested via comparison of nested models; the model with interactions was kept if the statistic of the test exceeded the fractile of order 1-*α* of a law of *χ*^2^. This method makes it possible to manually remove non-significant variables from the model after automatic selection of the variables while verifying that this does not significantly modify the parameters of the model. The comparison of nested models was tested by the maximum likelihood method at an *α* risk of 5%. The results of the multivariate analyses were presented as the crude odds ratio and adjusted odds ratio with a 95% confidence interval using the package Rcmdr implemented in R.

#### Semi-variogram and kriging interpolation for fish-borne trematode infection

The kriging method is widely used for predicting spatial patterns (e.g. the prevalence or intensity of infection) at non-sampling locations [25–29]. Kriging involves including a fixed number of nearest neighbour points within a fixed radius [26] and relies on semi-variograms that quantify spatial autocorrelation among all pairs of data according to distance [30].

Semi-variograms were estimated from survey data by calculating the squared difference of fluke intensity between all pairs of households. Intensity values were log + 1 transformed in order to fit the normal distribution. Semi-variance values were grouped and averaged according to separation distance (lags). A semi-variogram model was fitted as a line through the plotted semi-variance values for each lag using the packages automap and gstat [31] implemented in R. Kriging was obtained using the function krige of the package gstat and was applied using the values of the semi-variogram analysis, which interpolated or smoothed estimates, based on values at neighbouring locations and parameters from the semi-variogram [32].

### Local participation in a parasitic control programme and health education

In March 2013, after parasitic investigation and medical treatment, local administrations, i.e. the PCU in collaboration with the SAO, conducted a health education campaign aiming to reduce intestinal parasitic infection in the area. The programme focused on fish-borne parasitic diseases, one of the most common parasitic infections in the village of Huay Muang (see ‘Results’ section below). The villagers participated in a 2-day health education event which aimed to decrease raw food consumption and inform people of the negative health effects of foodborne parasitic infections and other intestinal parasitic diseases. The PCU and SAO also organised a cooking contest among the participants to create properly cooked traditional dishes with local freshwater fish under the motto ‘Cooked fish, Clean and Safe’.

Intestinal helminthic infection was reinvestigated in August 2013, after medical treatment and health education, again using a modified Katz thick smear technique (described above). The faecal examination was conducted by a local health service, the parasitological laboratory of Tha Wang Pha District Hospital. The participants, including those who had initially tested positive for intestinal parasites (i.e. paragonomiasis, fish-borne trematode and taeniasis patients) were also invited for a group discussion after taking part in the health education programme. Intestinal helminthic infection pre- and post-medical treatment and health education were compared and further discussed.

## Results

### Socio-demographics, customs and food consumption of Huay Muang villagers

Of the 228 participants (50.6% participation rate) for whom parasitic data were acquired, demographic and social characteristics were available for 198 individuals (except for sex and age, for which data were available for all 228 participants from PCU records). Gender participation was balanced (49.6% males and 50.4% females). The level of education was rather low, with the majority of participants having no education or primary school education only (87.4%). The main employment of participants was related to rice and maize plantations (69.7%), although this mainly applied to a considerably older age group (Table 1).

**Table 1.** Demographic and social characteristics of participants to questionnaires and parasitological survey in Huay Muang village

Variables	Number	Percentage (%) (IC 95%)
Gender: male/female	113/115	49.6 (43.1–56.1)/50.4 (43.9–57)
Age: <30 years	51	22.4 (16.6–28.2)
Between 30 and 50 years	93	40.8 (34–47.6)
>50 years	84	36.8 (30.5–43.1)
Education: no	73	36.9 (30.2–43.6)
Yes: primary school	100	50.5 (43.5–57.5)
Yes: high school and technical college	25	12.6 (7–17.2)
Occupation		
Unemployment	26	13.1 (8.4–17.8)
Planter (maize)	106	53.5 (46.6–60.4)
Farmer (rice fields)	32	16.2 (11.1–21.3)
Others (students, sellers…)	34	17.2 (11.9–22.5)
Visit physician if ill: yes/no	150/48	75.8 (69.8–81.8)/24.2 (18.2–30.2)
Screening parasites last year: no	164	82.8 (77.5–88.1)
Yes with negative results	22	11.1 (6.7–15.5)
Yes with positive results	12	6.1 (2.8–9.4)
Drink alcohol daily: yes/no	99/90	50.0 (43–57)/50.0 (43–57)
Use toilet: yes/no	152/46	76.8 (71–82.7)/23.2 (17.3–29.1)
Animals: no	10	5.05 (2–8.1)
Yes: pets (cats and dogs)	21	10.6 (6.3–14.9)
Yes: livestock (chickens, pigs, cows)	167	84.3 (79.2–89.4)

In terms of access to health services, the majority of participants asked advice from a doctor in case of illness in the previous year (75.8%). However, most (82.8%) did not examine their stools to check for the presence of parasites.

Regarding eating habits, 74.7% of the population consumed uncooked fish in traditional dishes, with a large proportion of the population (82.3%) obtained fish from the natural environment (rivers or ponds) (Table 2). A large majority of individuals (73.7%) also consumed raw meat; 8% consumed raw pork and 77.4% consumed raw beef. The source of wild meat was reported as personal hunting activities (46.6%) or neighbours (61.1%).

**Table 2.** Food habits of participants to questionnaires of Huay Muang village

Food habits	Number	Percentage (%) (IC 95%)
Raw fish consumption: yes/no	148/50	74.7 (68.6–80.8)/25.3 (19.2–31.4)
Wild fish: yes/no	163/35	82.3 (76.9–87.6)/17.7 (12.4–23)
Fish from rice fields or ponds: yes/no	6/192	3.03 (0.64–5.4)/97.0 (94.6–99.3)
Fish from market: yes/no	127/71	64.1 (57.4–70.8)/35.9 (29.2–42.6)
Raw meat consumption: yes/no	146/52	73.7 (67.6–79.8)/26.3 (20.1–32.4)
Beef consumption: yes/no	113/33	77.4 (71.6–83.2)/22.6 (16.8–28.4)
Buffalo consumption: yes/no	102/44	69.9 (63.5–76.3)/30.1 (23.7–36.5)
Pork consumption: yes/no	137/9	93.8 (90.4–97.2)/16.2 (11.1–21.3)
Wild animals consumption: yes/no	68/78	46.6 (39.7–53.5)/53.4 (46.5–60.3)
Meat supplied by: themselves/neighbours	121/77	61.1 (54.3–67.9)/38.9 (32.1–45.7)
Meat from market: yes/no	173/25	87.3 (82.7–91.9)/10.8 (6.5–15.1)

### Knowledge of parasites

Participants in the study were also questioned on their knowledge of the main types of parasites and their modes of transmission. Knowledge of what can be identified as a worm varied from 15.7% to 71.2% (Table 3). Regarding parasitic transmission routes, 90% of people were aware of the risks of ingestion of raw food, including fish, crabs and meat. More than 60% of respondents mentioned other routes of exposure such as not wearing shoes, handling food with dirty hands, consuming unfiltered or unboiled natural water, or the presence of flies on food (Table 3).

**Table 3.** Local knowledge of different types of parasites and their transmission ways

Questions	Number	Percentage (%) (IC 95%)
Do you know this helminth?		
Pinworms: yes/no	31/167	15.7 (10.6–20.8)/84.3 (79.2–89.4)
*Ascaris*: yes/no	118/80	59.6 (52.8–66.4)/40.4 (33.6–47.2)
Hookworms: yes/no	44/154	22.2 (16.4–28)/77.8 (72–83.6)
*Trichuris*: yes/no	31/167	15.7 (10.6–20.8)/84.3 (79.2–89.4)
Cestode: yes/no	141/57	71.2 (64.9–77.5)/28.8 (22.5–35.1)
Threadworm: yes/no	86/112	43.4 (36.5–50.3)/56.6 (49.7–63.5)
*Opistorchis*: yes/no	59/139	29.8 (23.4–36.2)/70.2 (63.9–76.6)
Do you know that you can be infected when:		
Walking barefoot: yes/no	127/71	64.1 (57.4–70.8)/35.9 (29.2–42.6)
Manipulating food with dirty hands: yes/no	139/59	70.2 (63.9–76.6)/29.8 (23.4–36.2)
Drinking unfiltered water: yes/no	126/72	63.6 (56.9–70.3)/36.4 (29.7–43.1)
There are flies over the food: yes/no	168/30	84.8 (79.8–89.8)/15.2 (10.2–20.2)
Consuming raw fish: yes/no	183/15	92.4 (88.7–96.1)/7.6 (3.9–11.3)
Consuming raw crab: yes/no	120/78	60.6 (53.8–67.4)/39.4 (32.6–46.2)
Consuming raw meat: yes/no	185/13	93.4 (90–96.9)/6.6 (3.1–10.1)
Consuming raw bush meat: yes/no	185/13	93.4 (90–96.9) /6.6 (3.1–10.1)

### Participatory mapping

**Fig. 1.**
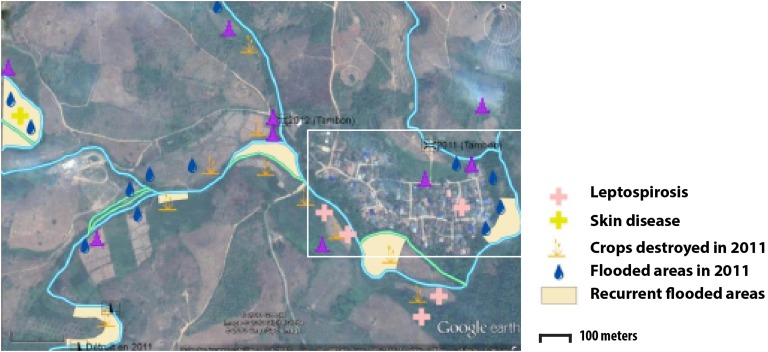
Results of the participatory mapping (see ‘Materials and methods’ section) showing the flooding areas and the unhealthy places according to risk perception of water-borne diseases.

Two types of flooding areas were mentioned by the participants: areas that flood regularly and areas flooded in 2011, a year characterised by a large flooding event that is still present in the memory of the population. Participants were aware of water-related contamination by several diseases such as leptospirosis or fish-borne parasitosis (Fig. 1). However, most of the flooding risks mentioned by the interviewees related to socio-economic risks associated with damage to crop fields. Thus, people are more concerned about risks threatening financial wellbeing than infectious diseases.

### Parasitological survey (pre-medical treatment/health education programme)

A parasitological survey of 228 individuals allowed us to detect nine species of intestinal parasites in the village of Huay Muang. Four types of helminth eggs and a larva were detected, including a cestode (*Taenia* eggs), two trematodes (*O. viverrini*-like eggs and *Paragonimus* eggs) and two nematodes (*S. stercoralis* and hookworm eggs). In addition, four protists were identified (*Blastocystis hominis*, *Endolimax nana*, *Entamoeba histolytica*/*dispar* and *Pentatrichonomas hominis*) (Table 4). The overall prevalence was 45.2% (*n* = 120) for helminth infections and 4.82% (*n* = 11) for protist infections. The dominant parasite eggs found in this study were the *O. viverrini*-like eggs (45.2%), followed by *S. stercoralis* (9.21%). All recorded intestinal protists showed a low prevalence (from 2.19% to 0.44%) (Table 4).

**Table 4.** Prevalence and intensity of helminths and protists in faecal samples obtained from Huay Muang village

Intestinal parasites	Pre-treatment/health educational programme			Post-treatment/health educational programme	
	Min-max (intensity±s.d.)	Number of infected people	Prevalence in % (CI 95%)	Number of infected people	Prevalence in % (CI 95%)
Helminths		120	45.2 (38.7–51.6)	10	4.4 (2.3–7.8)
Hookworms	0–12 (0.09±0.29)	4	1.75 (0.05–3.5)	0	0
Fish-borne trematode	0–503 (8.4±48.2)	103	45.2 (38.7–51.6)	6	2.6 (1.1–5.6)
*Paragonimus* sp.		4	1.75 (0.05–3.5)	0	0
*Strongyloides stercoralis*		21	9.21 (5.5–13.0)	2	0.9 (0.1–3.2)
*Taenia* sp.		11	4.82 (2.0–7.6)	2	0.9 (0.1–3.2)
Protists		11	4.82 (2.0–7.6)	N/A	N/A
*Blastocystis hominis*	0–4 (0.04±0.89)	5	2.19 (0.3–4.1)	N/A	N/A
*Endolimax nana*		*5*	2.19 (0.3–4.1)	N/A	N/A
*Entamoeba histolytica/E. dispar*		1	0.44 (−0.4 to 1.3)	N/A	N/A
*Pentatrichomonas hominis*		1	0.44 (0.04±0.89)	N/A	N/A

### Results of multivariate analyses

**Fig. 2.**
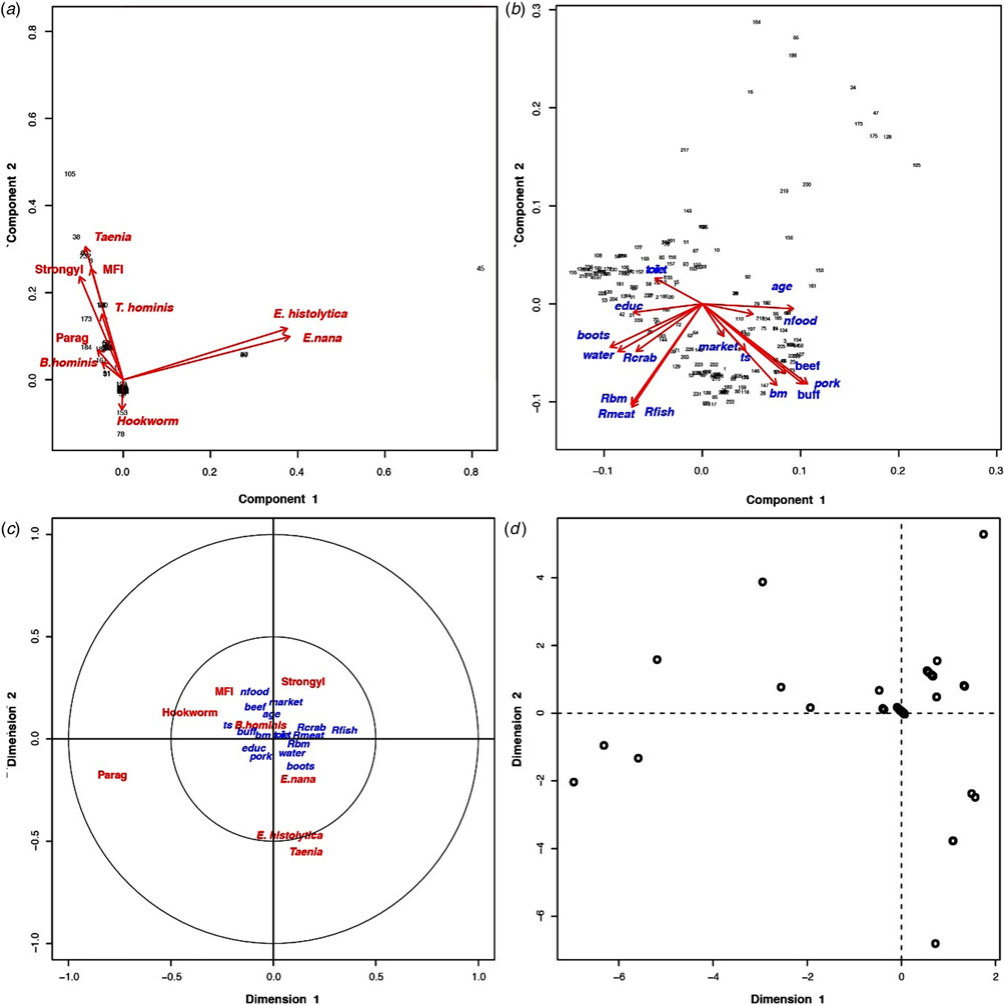
(a) Principal component analysis of parasites and their distribution among the inhabitants of Huay Muang, with the two first axes of the PCA explaining 31.3% of the variance. (b) Principal component analysis of the socio-demography and perceptions distribution among the inhabitants of Huay Muang, with the two first axes of the PCA explaining 45.9% of the variance. (c) Biplot graph of canonical correspondence analysis showing the association between intestinal parasites in relation to socio-demography (represented by PCA in (a)) and perceptions (represented by PCA in (b)) of Huay Muang villagers, with (d) the distribution of the inhabitants of Huay Muang. Rbm, raw bushmeat; Rmeat, raw meat (pork and beef); Rcrab, raw crab; Rfish, raw fish; water, water filtrated; boots, use of boots; market, food is mainly obtained from the market; ts, food is mainly obtained by the household; bm, consumption of bushmeat; pork, consumption of pork meat; buff, consumption of buffalo meat; beef, consumption of beef meet; nfood, consumption of natural food; toilet, presence of toilets; educ, level of education (from none to technical college); age, age; Strongyl, *Strongyloides* sp.; Parag, *Paragonimus* sp.; MIF, fish-borne trematodes.

**Fig. 3.**
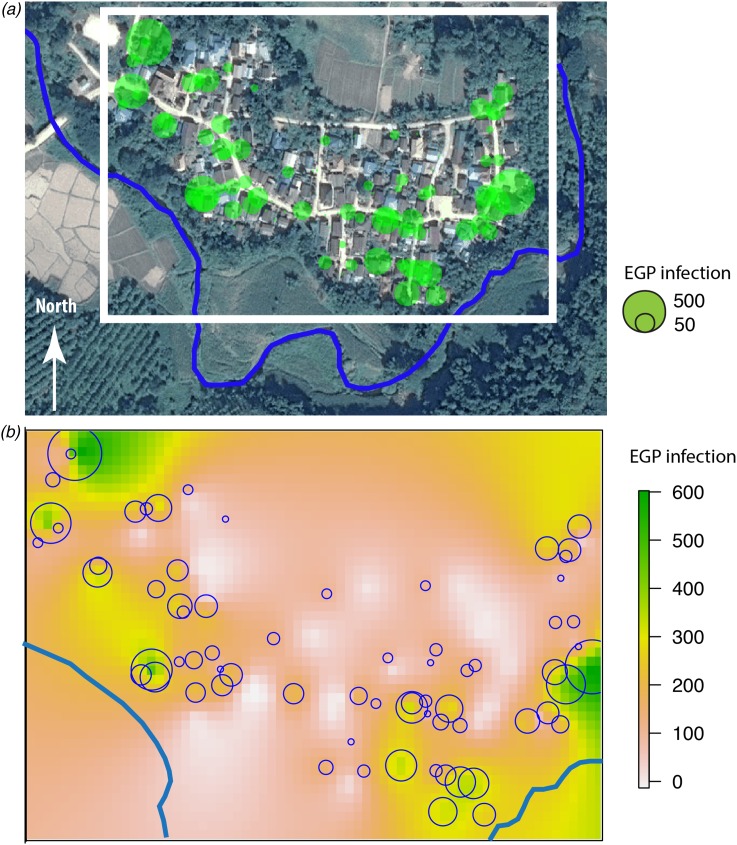
(a) Huay Muang map of fish-borne trematodes infection level (EPG) at household level with a size of green circles corresponding to the level of EPG infection for each household (white rectangle corresponds to the kriging area of (b); river in blue); (b) interpolation of fish-borne trematodes infection by kriging in a grid based on geographical coordinates of households with a scale of EPG infection from low infection in yellow to high infection in green.

Based on the PCA results, the villagers of Huay Muang appeared very dissimilar in terms of level and diversity of infection, with intestinal helminths and protists with a low proportion of variance of 16.9% for the first principal component and of 14.4% for the second principal component (Fig. 2a). Higher homogeneity was observed in socio-economics and/or customs based on questionnaire responses, with a proportion of variance of 25.4% for the first principal component and of 19.5% for the second principal component (Fig. 2b). Parasite infection was significantly associated with socio-economics, but with only the first canonical dimension being significant (Wilk's L = 0.37, correlation coefficient = 0.40; *P* = 0.04, Fig. 2c) (distribution of individuals is given in Fig. 3d).

### Results of univariate analyses

Univariate analyses indicated that factors related to an increase of infection load for an *α* risk of 20% were being over 30 years old, being male, owning domestic animals, eating raw fish or raw meat, fishing and hunting, and living close to the river. Factors that seemed to protect against infection were education (primary level or higher), the use of toilets, visiting a medical doctor, and knowledge of parasites and their transmission (Table 3).

### Determinants of fish-borne trematode intensity infection

The identified risk factors for infection with a fish-borne trematode are presented in Table 5. The rate of infection with fish-borne trematodes was significantly higher among people over 50 years of age compared with those under 30 (*P* = 0.009, Table 5). The intensity of infection (i.e. the number of parasite eggs) in people consuming raw fish was 10 times the intensity of infection in people that did not eat raw fish (*P* < 0.0001, Table 5). People's occupation played a role in the intensity of infection; being a corn planter seems to increase infection burden (odds ratio = 2.85, *P* = 0.04), while farmers appeared to be infected at a lower rate than unemployed people. Although not individually significant, these variables contributed to the selection of the best model (using the AIC value) (Table 5).

**Table 5.** Identification of risk factors influencing infection of fish-borne trematodes in Huay Muang village

Variables	Positive number (for a total=194)	Crude odds ratio	Estimated coefficient	Adjusted odds ratio	IC 95%	*P*-value
Age; <30 years *vs*	11					
Between 30 and 50 years	40	2.4	0.23	1.26	(0.40–3.53)	0.703
More than 50 years	50	10.6	1.95	7.01	(2.19–20.3)	0.0009
Consumption of undercooked fish No *vs*	9					
Yes	78	11.6	2.31	10.1	(3.85–267.0)	< 0.0001
Occupation: unemployed *vs*	13					
Rice farmers	15	1.4	−0.63	0.53	(0.15–1.78)	0.293
Planters of maize	55	3.8	1.05	2.85	(0.9188–78.89)	0.043
Others	4	0.2	−0.23	0.79	(0.16–3.61)	0.780
Screening of parasites previous year: no *vs*	73					
Yes, negative	4	0.09	−3.43	0.03	(0.01–0.13)	< 0.0001
Yes, positive	10	0.42	−0.73	0.48	(0.14–2.15)	0.256
Distance to river: >100 m *vs*	15					
<60 m	42	16.1	2.09	8.10	(3.07–21.2)	< 0.0001
>60 and <100 m	30	7.5	1.73	5.65	(2.31–13.4)	<0.0001

Finally, the distance from a person's house to the river was significantly associated with an increase in the intensity of infection by fish-borne trematodes. People living within 60 m of the river, i.e. in the row of houses closest to the river, had an infection intensity nine times that of people living further than 100 m from the river, whereas people living between 60 and 100 m of the river, i.e. in houses of intermediate distance from the river, had an infection intensity 5.6 times that of people living further than 100 m from the river, i.e. in the houses furthest from the river (Table 5).

### Semi-variogram and kriging of fish-borne trematode

The spatial distribution of the fish-borne trematode among households (Fig. 3a) was analysed using semi-variogram analysis with the best model function spherical. The kriging interpolation, using the results of the semi-variogram analysis, is represented in Figure 3b. A high predicted density of fish-borne trematode infection corresponded to recurrent flooding areas identified by participants during participatory mapping (Fig. 1).

### Helminth infection after health education

All participants (*n* = 228) who sent faecal samples for the first parasitic investigation (in November 2012) also submitted faecal samples for re-examination after medical treatment and health education (in May 2013). Total helminth infection rates sharply reduced from 45.2% to 4.4% between the first and the second investigation. The infection rate of *O. viverrini*-like eggs was also reduced, including eggs of fish-borne trematodes (from 45.2% to 2.6%), *S. stercoralis* (from 9.2% to 0.9%) and *Taenia* sp. (from 4.8% to 0.9%). *Paragonimus* sp. and hookworms were not detected at all in the latter examination. Selected helminth-infected patients were asked to attend a more in-depth interview to study their opinions about food consumption before and after health education (Table 6).

**Table 6.** Comparison of interviewing information from helminth-infected patients before and after receiving health educational activities (extracted from the report of the Primary Care Unit to the Subdistrict Administrative Organization)

Group	Before implementation	After implementation
Paragonimiasis patients	Patients usually catch fresh water crabs and shrimps from stream and river for cooking, e.g. grilling, burning, dropping in boiled water or making curry, which may not be properly cooked. They also like to bring shrimps from the fresh water sources to do the traditional raw dishes.	Patients realised that they will be infected by parasites if they consume raw crabs, shrimps, fishes or shellfishes. They will not eat them raw, and they need to be cooked before eating.
Fish-borne trematodes-infected patients	Patients like to bring several species of cyprinoid fishes, e.g., Javean barb, Silver barb, Goldfin tinfoil barb and small-scale mud carp to make a raw dish called ‘laab pla’.	Patients say that they better cook fish rather than eat it raw. Proper cooked food is clean and safe.
Taeniasis patients	Patients like to eat raw pork or beef. It is also found that those who eat raw meat and are infected with parasites usually do self-medication by taking a dose of deworming drug (Niclosamide) every 6 months.	Patients say that now they do not like eating raw meat but prefer proper-cooked meal instead.
In general	Some patients realised the effects of parasites on their health. However, in some situation such as going to work in distant field, they may be willing to take risk, e.g. drinking water from stream or pond; walking barefoot in the field because it is more convenient. Because of personal craving to eat raw fish or meat, just a little bit (not eat much), then they thought that it will not be contaminated by parasites.	Now, villagers are afraid of parasites. So, they are informed and avoid any risk of exposure to parasitic infection, such as eating raw food, drinking water from natural sources without boiling or filtering and not wearing shoes when working in the field.

## Discussion

The present study integrates several aspects of knowledge regarding parasites and their transmission to determine health risk factors based on a parasitological survey in a rural community. The parasitological survey showed that the most prevalent intestinal parasite found in human stools was fish-borne trematodes (45.2%). Although *S. stercoralis* (9.21%) and hookworms (1.75%) were present, no other soil-transmitted helminths were found despite the fact that 59.6% of the participants indicated that they were aware of *Ascaris lumbricoides.* This nematode was surely present in the past and may have disappeared or may still circulate at a low transmission rate in the community, or/and its symptoms may be more commonly observed in children than adults [33]. *Strongyloides stercoralis* is highly endemic to Southeast Asia and has been reported in 32 community-based studies in Thailand (overall prevalence of 23.7%) [34]. According to a previous report [34], this strongylid nematode has a very low prevalence in communities where faecal contamination is rare. The lack of sanitation reported by several households, in addition to the custom of walking barefoot, are factors favouring its transmission despite the fact that the majority of participants are knowledgeable about its transmission. The presence of hookworms was low, 10.2% in the present study, unlike in other recent surveys in rural Thai communities, in which prevalence ranged from 4.4% to 20.7% [11, 35]. It has been shown that walking barefoot increased the risk of hookworm transmission by 2.8 times in central Thailand [11]. In our study, 64.1% of participants were aware that walking barefoot increases the risk of parasite transmission.

The presence of *Paragonimus* in the studied population (1.75%) is of interest as information about this parasite in humans is limited to some occasional cases of paragonimiasis reported in remote hilly areas of Thailand [36]. Despite the fact that 92.4% of participants were aware that consuming raw crab meat can transmit parasites (by unintentional ingestion of metacercariae), the presence of four cases in the studied population showed that culinary habits prevailed over the perceived health risk.

The presence of *Taenia* eggs (taeniasis) in stools (4.82%) may be associated with the consumption of unregulated meat (pork and beef). No veterinary services control for the presence of *T*. *asiatica*, *T*. *solium* or *T*. *saginata* in Thailand [37]. According to the questionnaire results, the consumption of wild animals (including wild boars) was high, with 46.6% of respondents mentioning the consumption of bushmeat, which could also be an additional source of taeniases [38]. Moreover, the consumption of raw meat (a custom followed by 73.7% of participants), including pork (93.8%) and beef (77.4%), carries other parasitological risks [38]. Similar reports have discussed the difficulty of reducing the prevalence of fish-borne trematode infection by reducing the consumption of raw fish, even with health campaigns [39]. The high prevalence of *O. viverrini*-like eggs (fish-borne trematodes) in faecal samples, 45.2%, highlights the fact that culinary culture plays an important role in parasite transmission even if the majority of the community is aware of the risks of eating raw fish (see below).

The prevalence of intestinal protists was low, varying from 0.44% to 2.19% depending on the species. The reported prevalence of *B. hominis* (2.19%) was related to the consumption of contaminated food or water, close contact with animals, and poor sanitation. The faecal–oral route is the major transmission mode of this protist [40]. It is frequently found in dogs [41] and is associated with gastrointestinal disorders [42]. It should be noted that the diagnostic technique for *Blastocystis* sp. used in the present survey has a lower sensitivity than other techniques [43]; consequently, this prevalence could be an underestimation. *Endolimax nana*, *Entamoeba spp.*, and *P. hominis* have similar transmission modes. Infection with *P*. *hominis* is considered to be asymptomatic, but this parasite is a probable causative agent of gastrointestinal disturbances in children [44], and again is frequently reported as highly prevalent in dogs [45]. In addition to the potential pathogenicity of these parasites, their presence is an indicator of poor sanitary conditions, given their faecal–oral transmission route [9].

The most prevalent and/or pathogenic parasites observed in the community are transmitted through culinary habits (fish-borne trematodes, *Paragonimus* and *Taenia*). Parasites transmitted through contaminated water, food and soil were less prevalent. Altogether, these observations show that while public health campaigns have succeeded in improving sanitation, they are less effective in influencing cultural culinary habits.

Canonical analysis confirmed a statistical association between intestinal parasites and high-risk behaviour across the population. In particular, the habit of eating raw fish appeared to explain the observed association.

The knowledge of participants concerning helminth parasites showed high variability according to helminth species. *Ascaris* and *Taenia* were the two most recognised species, possibly because the adult forms of these parasites are expelled in faeces after self-medication (deworming drugs purchased from the local pharmacy) and are bigger than other helminth species. It is less common to recover *Trichuris* and fish-borne trematodes in faeces, which may explain the lower level of recognition. Few studies have attempted to study the ability of rural communities to identify helminth species in Asia compared with other geographical areas such as Kenya [46], Tanzania [47] or the Ivory Coast [48]. One study performed in Bangladesh [49] was limited to soil-transmitted helminths and did not provide many details about the helminths identified by participants. In Southeast Asia, previous studies on local knowledge were limited to *O. viverrini* [39]. The present study is one of the few that investigate local parasitological knowledge; however, this should be extended to other communities in Thailand and across Southeast Asia. This study is thus in line with the commitment of the NHA to bring evidence and knowledge into the sphere of health policy-making [50].

The responses to questionnaires show that villagers possess a good level of knowledge concerning the routes of parasite transmission, such as the consumption of raw crab, raw fish and raw meat, identified by more than 90% of the participants. Despite this, the inhabitants did not act accordingly, as 74.7% reported consuming raw fish regardless [39]. A similar pattern was observed in the consumption of raw meat despite the associated risks, which the local community is also aware of. Although cysticercosis is a serious parasitic disease, the results of the questionnaires highlighted the difficulties associated with reducing raw pork consumption, a deeply rooted custom in northern rural communities in Thailand [38]. The consumption of bushmeat by 46.6% of participants is also an important risk factor for several zoonotic infections [51, 52].

The results of principal component analyses showed a heterogeneous distribution of both intestinal parasites and high-risk behaviour across the community. This may reveal a greater heterogeneity of the population with respect to their wealth and parasitic infections compared with less developed rural areas of Southeast Asia [20]. The results of canonical analysis confirmed that the variability in parasite infection among inhabitants was related to the variability of socio-economic factors and associated behaviours. As lifestyles evolve, average wealth is improving along with health [14, 53]. Health information campaigns and the improvement of the health care system have positively impacted parasitological burdens, although social and economic gaps are widening.

The majority of social-demographic factors associated with infection with fish-borne trematodes are highlighted, with the exception of poor hygiene. As already mentioned, the most prevalent parasite was *O. viverrini*-like eggs, or fish-borne trematodes (probably minute intestinal flukes, e.g. *Haplorchis taichui*), which are recognised as a public health problem in northern and northeastern Thailand as well as some other countries in Southeast Asia [2]. In Northern Thailand, particularly in Nan Province, it seems that minute intestinal flukes are more common than the liver fluke *O. viverrini* [13, 54]. A very high incidence of the minute intestinal fluke *H. taichui* was reported in a study area in Nan Province (Chalerm Phrakiet District), but no *O. viverrini* infections were detected [55]. Similarly, it was confirmed in a study in Nan Province (Bo Kleu District) that no *O. viverrini* adult worms were recovered after treatment of suspected opisthorchiasis cases; all of those worms were *H. taichui* [54]. In addition, a high prevalence of *H. taichui* was shown both in intermediate hosts (snails and fishes) and villagers within the same area of Nan Province (Bo Kleu and Pua Districts) [56]. However, this does not rule out the possibility that cases of *O. viverrini* infection exist in the study area. Fish-borne trematodes still require further genetic identification to confirm whether they are *O. viverrini* or some other minute intestinal flukes (Heterophyidae and Lecithodendriidae), as the eggs of these flukes are practically impossible to differentiate morphologically to the species level [57, 58]. Metacercariae in fish are the source of human trematode infection; the local habit of eating raw fish was investigated in order to implement better preventive strategies and control transmission [39]. The maximum number of eggs per gram of faecal matter (EPG) detected in an individual in the studied community was 503 EPG. According to another researcher, the intensity of infection may be classified as mild (1–999 EPG), moderate (1000–9999 EPG) or high (⩾10 000 EPG) [59]. The average class of infection in the present study community was mild, but with a high prevalence (>40%), a pattern which has been observed previously [60].

Significantly higher infection intensities were observed in participants who mentioned the consumption of raw fish in the weeks prior to the study, which primarily included people aged over 50 years. According to their responses, people in this age group did not use deworming drugs.

Our results showed that there was a significant negative association between the proximity of houses to the river and infection intensity of fish-borne trematodes. This result supports previous findings [61] but here was observed at a high resolution, i.e. at the household level. The spatial density of fish-borne trematode infections predicted by kriging analysis seems to correspond to recurrent flooding areas mapped by participants during the participatory mapping. However, we should be aware of the limitations of our study, which was conducted at a small scale (households in one village) with the assumption of a spatial continuity in the infection process (the distance to river and flooding areas). Investigation of other villages is needed to confirm that kriging analysis is applicable to the investigation of parasitic infections on a small scale. We did not have information regarding the amount of fish consumed per habitant, nor on the relationship between this amount and direct access to the river, nor the sharing of raw fish dishes among neighbours (which is considered to be a risk factor) [10]. We did not find a relationship between the use of toilets and fish-borne trematode infection as observed in several previous studies [61, 62]. Indeed, a large majority of participants reported that they use toilets. Wastewater may contribute to contamination of the river during times of heavy rain, particularly in plots of land located near the river, where recurrent flooding was recorded by villagers. However, the true boundaries of the flooding areas are hard to delimit without the use of external information, such as remote-sensing data. We found associations between higher intensities of MIF and short distances to the river, which roughly corresponded to flooded areas according to the participatory mapping. The participatory mapping suggested a spatial association between flooded areas and higher intensities of fish-borne trematodes. One hypothesis is that flooding events may wash eggs onto flooded plots, which may facilitate maintenance of the parasite lifecycle. The nearby residents and owners of these flooded plots may then risk infection by eating the fish that populate their land.

The importance of a better understanding of the socio-economic aspects of this issue has been highlighted by another researcher [63]. Action taken by local public health administrative offices is key to sustainably controlling infectious diseases, including intestinal parasites. The 2014 NHA resolution insisted on the need to involve communities and to develop study plans for schools to educate pupils about safe culinary habits. A health education programme organised by the subdistrict PCU and SAO was positively received by the villagers. The participants acquired more knowledge of intestinal parasite infections, which in turn improved their culinary behaviour, sanitation and hygienic practices. The highlights of the health education programme were: (1) to translate the research outcomes to the local community; (2) to provide knowledge about endemic parasites in the area, their life cycles and their effects on human health; (3) to encourage better eating habits by avoiding raw or undercooked meat, which was expected to solve or reduce the problem of intestinal parasitic infections. However, annual monitoring of intestinal parasitic infections is required to assess changes in infection status and thus the efficiency of the implemented health education programme.

## Conclusions

Despite an accurate knowledge of worms and their transmission, particularly through traditional consumption of raw fish and raw meat, the studied rural community harboured some parasitic infections of significance. Our study emphasises the importance of studying the perceptions and practices of local communities in order to improve disease control strategies and public health. [64]. Moreover, our study shows that participatory methods, from mapping to health education, offer great opportunities for ecological epidemiology.
